# An adaptive phase II/III safety and efficacy randomized controlled trial of single day or three-day fixed-dose albendazole-ivermectin co-formulation versus albendazole for the treatment of *Trichuris trichiura* and other STH infections. ALIVE trial protocol

**DOI:** 10.12688/gatesopenres.13615.1

**Published:** 2022-05-05

**Authors:** Alejandro Krolewiecki, Wendemagegn Enbiale, Javier Gandasegui, Lisette van Lieshout, Stella Kepha, Augusto Messa Junior, Michel Bengtson, Woyneshet Gelaye, Valdemiro Escola, María Martinez-Valladares, María Cambra-Pellejà, Jaime Algorta, Helena Martí-Soler, Pedro Fleitas, Maria Rosa Ballester, Stephen R. Doyle, Nana Aba Williams, Almudena Legarda, Inácio Mandomando, Charles Mwandawiro, José Muñoz

**Affiliations:** 1 Barcelona Institute for Global Health (ISGlobal), Hospital Clínic—Universitat de Barcelona, Barcelona, Spain; 2 Instituto de Investigaciones de Enfermedades Tropicales, Universidad Nacional de Salta, Oran, Salta, 4530, Argentina; 3 College of Medicine and Health Science, Bahir Dar University, Bahir Dar, Ethiopia; 4 Department of Dermatology,, Amsterdam Institute for Infection and Immunity, Academic Medical Centre, Amsterdam, The Netherlands; 5 Department of Parasitology, Centre of Infectious Diseases, Leiden University Medical Centre (LUMC), Leiden, The Netherlands; 6 Eastern and Southern Africa Centre of International Parasite Control, Kenya Medical Research Institute (KEMRI), Nairobi, Kenya; 7 Centro de Investigação em Saúde de Manhiça (CISM), Maputo, Mozambique; 8 Instituto de Ganadería de Montaña, CSIC-Universidad de León, Grulleros, León, Spain; 9 Departamento de Sanidad Animal, Facultad de Veterinaria, Universidad de León, Campus de Vegazana, León, Spain; 10 Liconsa SA, Azuqueca de Henares, Guadalajara, Spain; 11 Institut d’Investigació Biomèdica Sant Pau (IIB SANT PAU), Barcelona, Spain; 12 Faculty of Health Sciences Blanquerna,, University Ramon Llull, Barcelona, Spain; 13 Centro de Investigación Biomédica en Red de Salud Mental (CIBERSAM), Barcelona, Spain; 14 Wellcome Sanger Institute, Hinxton, Cambridgeshire, UK

**Keywords:** Antihelmintics, STH, Trichuris trichiura, Strongyloides, Hookworm, Albendazole, Ivermectin

## Abstract

**Background: **Soil-transmitted helminths (STH) are targeted for control through mass drug-administration campaigns to prevent morbidity affecting at-risk groups in endemic regions. Although broadly successful, the use of albendazole and mebendazole achieved variable progress, with deficiencies against *Trichuris trichiura* and a predictable low efficacy against *Strongyloides stercoralis*. Novel drug combinations offer a potential solution, providing they can be delivered safely and maintain efficacy against all STH species. Here we present the protocol of a clinical trial to evaluate a fixed-dose combination (FDC) tablet containing albendazole and ivermectin that will be compared against albendazole against STH *.*

**Methods: **An adaptive phase II/III randomized controlled trial will be undertaken in STH endemic sites in Ethiopia, Kenya and Mozambique to evaluate an oral FDC of 400 mg albendazole and either 9- or 18 mg ivermectin. FDC will be administered as a single dose or single doses over three-consecutive days and assessed against a single dose of 400 mg albendazole. In the phase II trial, 126 *T. trichiura*-infected children weighting 15 to 45 kg will be treated in a dose-escalation manner to determine safety objectives. In the phase III trial, 1097 participants aged 5 to 18 years old infected with *T. trichiura, *hookworm and *S. stercoralis *will be recruited to determine safety and efficacy. The trial will be open-label with blinded outcome assessors. Cure rate measured 21-days after-treatment in duplicate Kato-Katz is the primary efficacy outcome. Secondary objectives include efficacy evaluation by quantitative polymerase chain reaction (PCR) as an outcome measurement, description of pharmacokinetic parameters, palatability and acceptability evaluations, and monitoring of anthelmintic resistance.

**Conclusions:** This trial with registrational goals seeks to evaluate an innovative fixed-dose combination of albendazole and ivermectin co-formulated tablets, with the goal of providing an anthelmintic regimen with improved efficacy and spectrum of coverage against STH.

**ClinicalTrials.gov registration:** NCT05124691 (18/11/2021).

## Introduction

Neglected tropical diseases (NTDs) are a group of infectious and non-infectious diseases that affect pediatric and adult populations in the world’s poorest communities ^1^. Among the NTDs that cause significant global morbidity and mortality, soil-transmitted helminths (STH) infections ( *Ascaris lumbricoides*, *Trichuris trichiura,* and the hookworms *Ancylostoma duodenale* and *Necator americanus*) are associated with malnutrition, impaired growth and cognitive development in children ^2^. It has been estimated that almost 900 million people were infected with at least one of these STH species in 2017, corresponding to 1.7 million years lived with disability (YLDs) attributable to STH infection ^3^. *Strongyloides stercoralis*, also a STH but with distinctive biological and life-history features, has been traditionally excluded from control initiatives but has recently been included in the World Health Organization (WHO) targets for 2030 ^4, 5^. It is estimated that over 350 million people are infected by *S. stercoralis* worldwide ^6^. The current WHO mandated control strategy for STH emphasizes morbidity control through mass drug administration (MDA) of benzimidazole-class anthelmintics – primarily albendazole (ALB) or mebendazole (MEB) – targeting pre-school and school-aged children (SAC), women of childbearing age and adults in certain high-risk occupations ^7^. Treatment with ALB or MEB is given as a single dose once or twice a year (depending on the estimated prevalence of STH in the community) and both have excellent safety profiles ^7^. With this approach, the global target is to achieve elimination of STH as a public health problem in 96% of the 101 currently endemic countries by 2030 ^8^.

While improvements in water and sanitation, and mass administration of benzimidazoles remain the cornerstone for reducing STH morbidity, there are increasing concerns that the ongoing success of these programs may be hindered, among other reasons, by the reliance on a single class of drug ^9^. A recent systematic review and network meta-analysis identified worrisome efficacy indicators against *T. trichiura* (30.7% CR, and 49.9% ERR), with even lower efficacy in more recent studies, falling from 38.6% in 1995 to 16.4% in 2015; for hookworm (79.5 CR and 89.6 ERR) the indicators were more satisfactory and for *A. lumbricoides* efficacy remained adequate (95.7 CR and 98.5 ERR) ^10^. Neither ABZ nor MEB in single doses have any significant activity against *S. stercoralis* ^4^. In parallel, there is growing concern that widespread monotherapy could lead to selection pressures resulting in the emergence of drug resistant populations of parasites surviving treatment. Although there is little evidence to suggest this is a problem for STH control now, there is clear and widespread evidence of drug resistance in helminths of veterinary importance; therefore, represents a credible risk to global STH control programs in the near future ^11^.

Considering the low efficacy of treatment against some STH species together with the risk of anthelmintic resistance by reliance on a monotherapy, a combination therapy using existing drugs with complementary but distinct modes of action is a potential solution. The combination of ALB and ivermectin (IVM) has been identified in different trials and systematic reviews as one that combines adequate safety, lack of significant drug-drug interactions and importantly, efficacy against all STH, including both *T. trichiura* and *S. stercoralis* ^12– 15^. Due to the different mechanisms of action of both drugs, this combination has a theoretical lower risk for the selection of resistant parasites than a single drug treatment.

In the search for safe, efficacious and logistically simple to distribute and administer therapeutic alternatives, the “Stop Transmission of intestinal Parasites” (STOP) Project Consortium has developed a public-private international collaboration for the design and validation of innovative products and strategies for the control of STH as a public health problem ^16^. The study protocol presented in this report summarizes the progress and achievements leading to the registrational clinical trial that aims to evaluate the efficacy and safety of an ALB-IVM fixed-dose combination (FDC), co-formulated for the treatment of STH.

### Study rationale and hypothesis

We hypothesize that a FDC of 400 mg ALB together with 9 mg or 18 mg IVM (the specific dose chosen according to body weight), as a single dose or three-day regimen, will be more effective against STH compared to the current strategy of a single dose of 400 mg ALB while maintaining an excellent safety profile. To test this hypothesis, we designed an adaptive phase II/III superiority trial (called the ALIVE trial) that will primarily target safety objectives in the phase II component and safety and efficacy in the phase III component.

**Figure 1.**
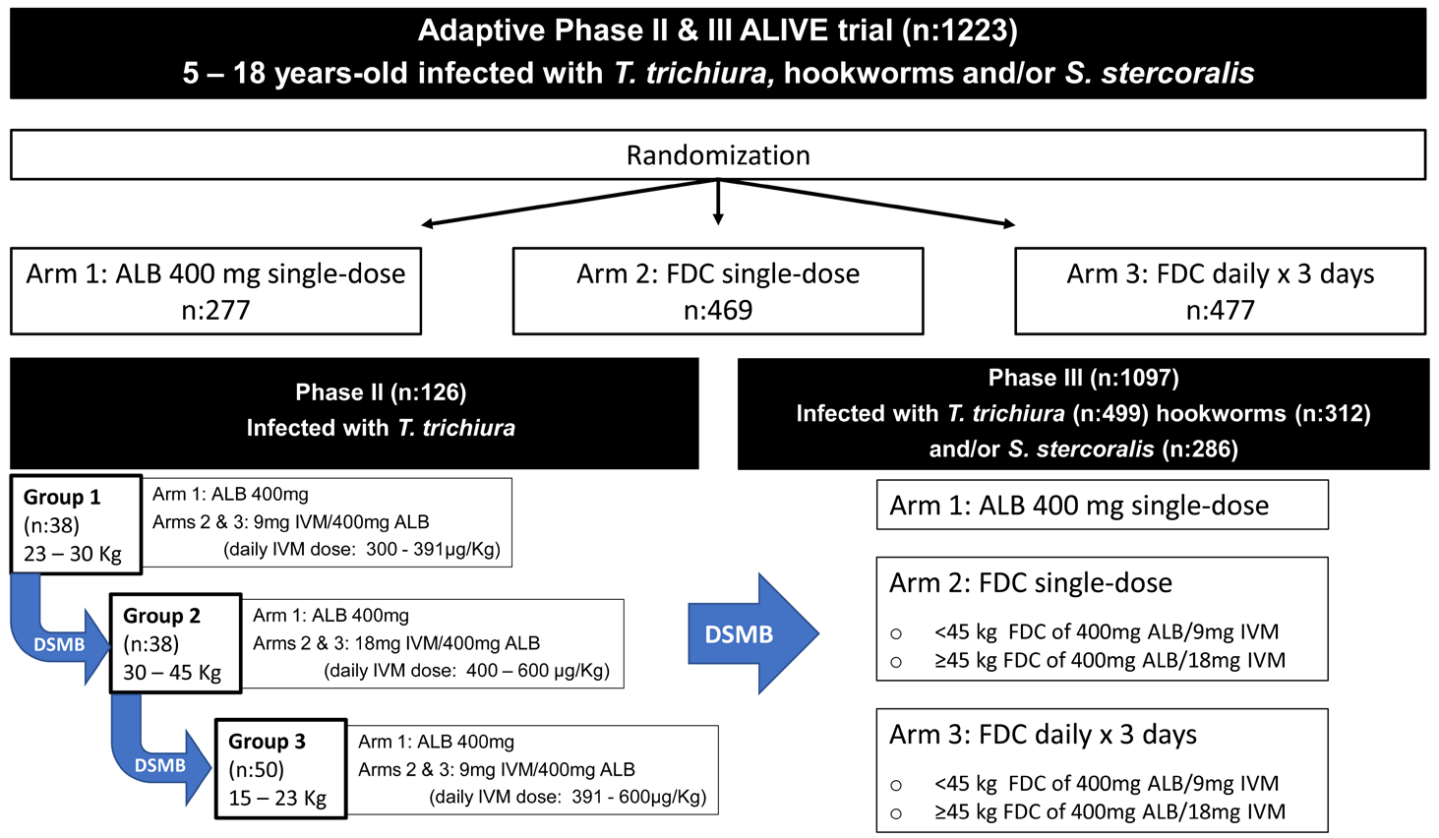
Flow diagram of the phase II & III trials, including participant sample size and randomization in three treatment study arms, dose escalation in phase II based on weight stratification, and targeted soil-transmitted helminth (STH) species in both trials. Abbreviations: ALB, albendazole; FDC, fixed-dose albendazole-ivermectin combination; DSMB, Data Safety Monitoring Board.

Despite the use of registered drugs for the medical indication under evaluation, the rationale for a phase II safety component is based on the use of a novel co-formulation and the consequent administration of a higher dose of IVM per kg than what is currently approved. These co-formulations consist of 9 mg or 18 mg IVM with 400 mg ALB; and with these two formulations trial participants between 15 and 90 kg of body weight would be treated with a dose of IVM between 200 μg/kg and 600 μg/kg ( Figure 1). Preliminary studies, including a systematic review and meta-analysis of the safety of high dose IVM, identified no concerns despite the limitations of the available data ^17^, and a study on high fixed-dose IVM in healthy adult volunteers contributed data on safety and pharmacokinetic parameters ^18^. Moreover, a trial of the co-administration of 400 mg ALB and 600 μg/kg IVM against *T. trichiura* infection in Honduran children reported non-significant safety findings (and a promising efficacy response) ^15^. Concerning this FDC, a study conducted in healthy adult volunteers with the aim of characterizing its pharmacokinetic profile, showed that the FDC was well tolerated, with no safety concerns (submitted for publication).

While the overall objective of the ALIVE trial is to identify a therapeutic approach with superior efficacy against STH as a group, the study design focuses on those species with suboptimal (hookworms), poor ( *T. trichiura*) or no ( *S. stercoralis*) responses to benzimidazoles in monotherapy but not on *A. lumbricoides*, which is currently adequately treated with ALB or MEB monotherapy ^19^.

## Protocol

Adaptive phase II/III randomized, superiority trial to compare the safety and efficacy of the active control arm (current standard of care, single dose of 400 mg ALB) against two experimental arms (single day or three-day single dose FDC of ALB+IVM) will be performed in pediatric and young adult populations in Bahir Dar (Ethiopia), Kwale (Kenya) and Manhiça (Mozambique).

### Study sites

In Kenya, the study will take place in Kwale county situated on the south coast, bordering Tanzania to the South-West. The total area of the county is approximately 8,270 km ^2^ with a population of about 760,897. The southern region of the county is on the shoreline of the Indian Ocean. The area has been identified as a hotspot for infections with *T. trichiura* and hookworms despite MDA campaigns through the National School-based Deworming Programme (NSBDP), active since 2012 ^20^. The full recruitment for the phase II trial will be performed at this site, as well as part of phase III.

In Ethiopia, the study will be conducted in the Bahir Dar Zurya woreda (district) at a height of 1,900 m above sea level, in the West Gojam zone of the Amhara region, which has a population of approximately 230,000 people. The district has 36 primary (grade 1 to 8) and six secondary (grade 9 and 10) schools. The student population is 49,013 with an average of 1,160 students in each school. The area has a high prevalence of hookworm and *S. stercoralis* infections ^21^.

The Manhiça district in Southern Mozambique, a peri-urban setting with a predominantly young population, will be the third site; it runs a Health and Demographic Surveillance System in the area which covers the entire district (2,380 km ^2^), with a population of approximately 204,000 inhabitants under surveillance, 44% of which are <15 years of age. Every household from the study area is geo-localized and each individual has a unique an identification number ^22^. The district has 93 primary and 9 secondary schools with a student population of 72,829. The area has been identified to harbor areas with significant prevalence of STH, including *S. stercoralis* ^23^.

### Objectives and endpoints

***Phase II component***. The phase II component of the trial will be held in Kwale, Kenya. The primary objective of this phase is an open label evaluation of the safety of the FDC as a single dose or a single dose delivered on three consecutive days for the treatment of *T. trichiura* in participants between 15 and 45 kg of body weight. Secondary objectives include: (i) measuring the efficacy of treatment against *T. trichiura* as well as other STH species in those subjects co-infected with the target parasites, (ii) the pharmacokinetics analysis, (iii) acceptability and palatability evaluations of the FDC. The efficacy data from the participants of the phase II component will be incorporated into the efficacy data of the phase III component for a joint analysis.

***Phase III component***. The phase III component of the trial will be held in all three study sites. The primary objective of the Phase III trial is to evaluate the efficacy assessed as CR rate at day-21 post-treatment by Kato-Katz of the above-mentioned experimental and control arms used in the phase II for the treatment of *T. trichiura* in pediatric and young adult populations. Secondary objectives include: (i) determining the CR against hookworms and *S. stercoralis* and ERRs of *T. trichiura* and hookworms determined by stool microscopy, (ii) evaluation by quantitative polymerase chain reaction (qPCR) of parasite specific DNA in calculating the primary outcome measurement (efficacy) compared to stool microscopy, and (iii) evaluation of the frequency of known ALB resistant alleles in hookworm and *T. trichiura* in the three treatment arms before and after treatment. Efficacy against *A. lumbricoides* is included as an exploratory objective. 

Reporting of the study protocol has been verified in accordance with the SPIRIT (Standard Protocol Items for Randomized Trials) recommendations ^24^.

### Trial design

***Phase II component***. The phase II component is a randomized, controlled, parallel-group, open-label (outcome assessor blinded), dose-escalation, trial. Participants (n=126) will be stratified by weight into three groups ( Figure 1). Within each weight group, there are three treatment arms, consisting of: (i) an active control arm receiving a single dose of 400 mg ALB, (ii) an experimental arm receiving a single dose FDC, and (iii) an experimental arm receiving three consecutive days of the single dose FDC. All participants will be allocated by simple randomization to one of the three study arms with unequal probability (ALB: 20%, FDCx1: 40%, and FDCx3: 40%). The randomization list will be generated using R software (RRID:SCR_001905), package blockrand ^25, 26^ by the trial statistician.

Progression to the next weight group will be granted by the Data Security and Monitoring Board (DSMB), provided the safety stopping rules are not met. The predefined stopping rules that would result in suspension of dose escalation in the FDC arm are the occurrence of ≥1 serious adverse reaction having a reasonable possibility of a causal relationship with the study drug in any of the weight groups in phase II and the overall assessment of the trial by the DSMB. Upon evaluation of the safety data of the whole phase II study population, the DSMB will assess clearance for the phase III component of the trial.

***Phase III component***. The phase III component is a randomized, multi-center, parallel-group, active-controlled, outcome assessor blinded (laboratory personnel performing Kato Katz and Baermann methods), superiority trial that will seek to recruit 1097 participants. These participants, together with the 126 participants of the phase II trial, bring the total to 1223 trial participants ( Figure 1).

The three treatments arms ( Figure 1) consist of:

A.Treatment Arm 1: Single dose tablet of 400 mg ALB (active control arm).

B. Treatment Arm 2: Single dose of the FDC tablet.

- For participants <45 kg of body weight at baseline: FDC of 400 mg ALB + 9 mg IVM
- For participants ≥45 kg of body weight at baseline: FDC of 400 mg ALB + 18 mg IVM

C. Treatment Arm 3: Daily dose of FDC tablet for three consecutive days:

- For participants <45 kg of body weight at baseline: FDC of 400 mg ALB + 9 mg IVM
- For participants ≥45 kg of body weight at baseline: FDC of 400 mg ALB + 18 mg IVM

Recruitment to both phase II and phase III components of the trial will be done at school and community levels through invitations by the local teams to group meetings, followed by individual meetings with parents willing to participate. Parents of children participating will be explained the process of informed consent, which will be completed by signing the informed consent form and written assent for participants 12 to 17 years old ( Table 1). Allocation of participants to the study arms will be done by block randomization and stratified by STH species. We will ensure balanced allocation to the three arms in the three study countries. The randomization list will be generated with the R software by the trial statistician ^25, 26^.

**Table 1.** Schedule of procedures for the phase II and III components of the ALIVE trial.

	Screening		Baseline	Follow-up				
Procedures	Pre-screening (up to -3 months)	Screening Day -7 to -1	Enrolment/ Baseline Day 0	Day 1	Day 2	Day 3	Day 7	Day 21+/- 7-day post treatment
Informed consent/assent	X	X						
Stool analysis (Baermann and Kato-Katz)		X						X
Inclusion and Exclusion criteria		X	X					
History & Physical exam		X	X					X
Urine Pregnancy test		X						
Randomization and treatment assignation			X					
Study drug administration single-day arms			X					
Study drug administration Arm 3 (FDCx3)			X	X	X			
Acceptability questionnaire single-day arms *			X					
Acceptability questionnaire Arm 3 (FDCx3) *					X			
Pharmacokinetics blood sampling * **			X	X	X			
Concomitant medication & disease review		X	X	X	X	X	X	X
Adverse event monitoring		X	X	X	X	X	X	X

Abbreviations: FDC, fixed-dose albendazole-ivermectin co-formulation. *: only for the phase II participants. **: Population pharmacokinetics: two timepoints per participant for ALB arm at 1, 2, 3, 4, 5, 6, 7, 8, or 24 h and for the single dose FDC arm at 1, 2, 3, 4, 5, 6, 7, 8, 24, 48 or 72 h; two timepoints for three-dose FDC arm at 1, 2, 3, 4, 5, 6, 7, 8, 24, 48 or 72h post-administration and one additional timepoint at pre-dose Day 3.

The main eligibility criteria include: (i) infections with *T. trichiura* for the phase II component and *T. trichiura*, hookworms and/or *S. stercoralis* for the phase III trial; (ii) participant age between 5 to 18 years-old, and (iii) participant weight ≥15 kg and < 45 kg for the phase II component specific weight strata are described in Figure 1. Exclusion criteria include: (i) treatment with benzimidazoles within the previous three months, (ii) pregnancy or first week postpartum, or (iii) the active use of warfarin.

### Diagnostics

At baseline and on day-21 (+/- 7 days), all participants will be asked to provide a stool sample. The sample will subsequently be divided into aliquots, which will be used for Kato-Katz thick smears, modified Baermann, qPCR (ethanol preserved), resistance testing and back-up storage. Duplicate Kato-Katz thick smears (41.7 mg each) will be prepared for examination under a microscope for eggs of *T. trichiura*, *A. lumbricoides* and hookworms by experienced technicians. For quality control, 10% of the slides will be randomly selected and re-read by an independent reader. Discrepant/inconsistent results will be considered; if there is a difference in presence/absence of a specific helminth species; or if differences in egg counts exceed 10 eggs for Kato-Katz slides with ≤100 eggs or exceed 20% for Kato-Katz slides with >100 eggs. If a discrepancy is detected between the first readings and the quality control (QC) reading, a third technician will read the slide and that reading will be considered the valid one. Modified Baermann will be performed with 3 grams of stool samples in all samples for identification of the presence of *S. stercoralis* larvae ^27^, and a second microscopist will be required to confirm the identification for QC.

Multiplex real-time quantitative PCR (qPCR) will be used for the detection of parasite-specific DNA in stool ^28^. The outcome of the qPCR is the cycle-threshold (C _t_)-value, which is the PCR cycle in which the level of fluorescent signal representing DNA amplification exceeds the background fluorescence; the C _t _value is inversely proportional to the DNA concentration of the target, and therefore, can be used to quantify the amount of parasite-specific DNA present in the sample tested. In addition, qPCR will allow the identification of hookworm species ( *N. americanus* and *A. duodenale*) to evaluate if there are differences in the response to treatment for these spe *cies.* Molecular diagnostic testing in the context of the ALIVE trial will include participation in the Helminth External Molecular External Assessment Scheme (HEMQAS) for quality assessment to ensure delivery of reliable and comparable results ^29^. For an extended description of all laboratory procedures and handling of samples, standard operating procedures (SOPs) of the ALIVE trial and STOP Consortium are publicly available at *https://stoptheworm.org/resources/protocols/* .

### Study medication

The FDC (400 mg ALB + 9 mg or 18 mg IVM) will be provided as an oro-dispersible and chewable tablet manufactured under Good Manufacturing Practices (GMP) by Laboratorios Liconsa SA (Guadalajara, Spain), which will be responsible for the pharmaceutical development and subsequent production, labeling, packaging and distribution to the trial sites; while ALB will be provided as a chewable tablet (Eskazole 400 mg tabets, Allen Pharmaceuticals, Spain). Both FDC and ALB will be taken after a light meal, followed by visual observation of the participant by the study physician at each site. A light meal is considered to be the equivalent of a soft breakfast with a contribution of 15 g of fat. Participants that vomit within one hour after treatment will require re-dosing. The participants will not be allowed more than one repeated dose. In case a participant vomits following redosing during the enrollment visit, the subject will be withdrawn from the trial and standard treatment will be offered according to national guidelines.

### Outcomes

***Safety***. Safety is the primary outcome in the phase II component. After signing informed consent, evaluations and measurements, including adverse events, vital signs, physical examination, weight, height and body mass index (BMI) will be assessed through post-treatment follow-up visit ( Table 1). All adverse events during all study visits, will be noted and surveillance 3 hours post-treatment will be conducted each day a participant receives study treatment. Type, frequency, duration, severity and relatedness to study drug will be recorded for every adverse event and severe adverse event. Any clinically significant abnormalities persisting at the end of the study will be followed up by the study physician until resolution or until a clinically stable endpoint is reached. Safety will also be evaluated as a secondary objective in the Phase III trial.

***Efficacy***. Efficacy estimated by CR for *T. trichiura* is the primary outcome of the phase III component. Anthelmintic efficacy, measured by CR for each STH target species, will be determined by comparative analysis of a stool sample taken before and 21 (+/-7) days after treatment by Kato Katz or the Baermann method ^19^. Cure is defined as absence of the STH species in participants who had a positive egg count and/or larvae count for that STH at baseline. CR is defined as the proportion of individuals cured to the total of those infected at baseline with each particular species of STH. 

Efficacy measured through ERR will be determined by using geometric means, calculated for hookworms, *T. trichiura* and *A. lumbricoides* from the findings on duplicate Kato-Katz samples taken both before treatment and again at day-21 post-treatment. The Baermann method will also be performed in parallel to Kato-Katz for the identification of *S. stercoralis*. CR and variation in C _t _values will be assessed by qPCR analysis between baseline and day-21 samples. All molecular testing in pre- and post-treatment samples will be performed blinded from the microscopy data.

***Pharmacokinetics***. Samples for population pharmacokinetic analysis will be collected by finger prick using Mitra sticks (Neoteryx, Toronto CA) (20 µL). After drying, the samples will be kept at room temperature for up to 21 days and at -80℃ until transport and processing at Kymos labs, Barcelona, Spain. Quantification of the study drugs will be performed by HPLC-MS/MS, with has a lower limit of detection of 5 ng/mL for IVM B1a and 5 ng/mL for ALB sulfoxide.

The 126 subjects included in the phase II trial will be randomly assigned to different sampling times (two blood samples for each participant in the single dose arms, plus a pre-dose on the 3 ^rd^ dose for arm three) to adjust a population pharmacokinetic model. A simultaneous pharmacokinetic analysis of all concentration-time data of either IVM or ALB will be performed by a nonlinear mixed-effects modeling approach.

***Participant acceptability/palatability***. The acceptability of the new FDC will be investigated in children during the phase II following standard procedures. For children 5-8 years-old, a Facial Hedonic Scale will be applied after drug administration. For older participants and parents, a Numerical Rating Scale, including specific questions on taste, smell, and texture, will be used.

***Resistance outcome***. We will evaluate genotypic resistance associated with treatment failures for *T. trichiura* and hookworms using genome-wide approaches on data which will primarily be generated by pooled sample whole-genome sequencing. By comparing genetic diversity analysis before and after treatment, we aim to identify genomic regions and genes under positive selection potentially due to ALB and/or FDC treatment. The second output will be a list of single nucleotide polymorphisms (SNPs) with statistical associations with treatment efficacy by both logistic and linear regression models. By these two outputs, we will define the genetics of treatment response and, therefore, potential markers of anthelmintic resistance in *T. trichiura* and hookworm. Unfortunately, we will exclude *S. stercoralis* from the resistance assessment due to the complexity of larvae culture and isolation for genomics analysis as previously described ^30^, which hamper its implementation in the context of a RCT.

### Data handling

Data will be collected in password protected electronic case report forms (eCRF) containing internal quality checks. Paper-based study visit worksheets for each participant containing all the relevant demographic and clinical data for the trial will be used as source documents. Clinical data will be entered from the source documents and no direct data entry in the eCRF will be performed. Laboratory results will be recorded in an *ad-hoc* database at each site. All data storage will be encrypted and password protected. All the data generated at the trial sites will run quality management procedures internally. Anomalies and missing data identified at the centralized database will be clarified and resolved with the site. Using SOPs developed *ad-hoc*, monitors will verify compliance with the approved protocol (including local regulations for each site) and with International Conference on Harmonization Good Clinical Practice (ICH GCP). Regulatory authorities and the sponsor will have access to all data for monitoring, auditing and inspection activities.

### Statistical methods

***Sample size calculation***. Sample size was calculated based on available data from peer-reviewed publications complemented with reasonable estimates of efficacy for those experimental groups that have not been previously tested. Given the public health relevance of generating data for each species of STH species of interest to this trial, sample size calculations are made to provide adequate power for each of these species, including that in the primary endpoint ( *T. trichiura*) and those in the secondary endpoints (hookworms and *S. stercoralis*). For these calculations, the efficacy of the control arm (400 mg ALB in a single dose), was obtained for *T. trichiura* and hookworms from a systematic review and meta-analysis where temporary trends in efficacy (with the corresponding confidence intervals (CIs)) were incorporated ( Table 2) ^10^.

**Table 2.** Sample size according to the expected efficacy (CR) of the different treatment. The estimated sample size was calculated for pairwise comparisons of the expected CR for three study groups with an overall significance level of 5% adjusted for multiple tests by Bonferroni's correction, and 80% power. The total sample size was calculated by inflating the estimated calculation by 10% considering losses to follow-up..

		Phase II *		Phase III		Phase II/III
Group	Expected Cure Rate (%)	N estimated	N total phase II	N estimated	N total phase III	N Total
***T. trichiura***						
ALB	23.7	23	26	93	103	129
FDCx1	43.7	45	50	178	198	248
FDCx3	59.0	45	50	178	198	248
**Total**		**113**	**126**	**449**	**499**	**625**
**Hookworms**						
ALB	79.5			91	101	101
FDCx1	79.5			91	101	101
FDCx3	95.0			99	110	110
**Total**				**281**	**312**	**312**
***S. stercoralis***						
ALB	45.0			42	47	47
FDCx1	79.0			108	120	120
FDCx3	94.0			107	119	119
**Total**				**257**	**286**	**286**
**Total trial**			**126**		**1097**	**1223**

*The phase II sample size corresponds to 20% of the total sample size for *T. trichiura*.

For the efficacy of the control arm against *S. stercoralis,* a clinical trial that included an arm of 400 mg ALB for three consecutive days was used, assuming a “best case scenario” for the efficacy of the control group. This estimated efficacy is also in the range of a systematic review assessing the efficacy of ALB at various (but not single) doses ^31^. For the FDC at single dose, the calculations were based on the estimated efficacies (and their corresponding CIs) in a systematic review that calculated the Relative Risks of cure of diverse drug regimens against a single 400 mg ALB dose ( Table 2) ^14^. For *S. stercoralis*, the estimated efficacy of FDC was calculated based on a recent clinical trial using IVM single and multiple-dose regimens ^32^. Finally, for the FDC in three-dose regimens, considering its use in public health, deployment logistics in MDA campaigns and expected impact of the FDC, we estimated that an improvement of at least 15 percentage points would be the minimum improvement in efficacy to be demonstrated in order to make the FDCx3 regimen worth considering in public health interventions. The sample size was calculated for pairwise comparisons of the expected CR for three study groups with an overall significance level of 5% adjusted for multiple tests by Bonferroni's correction, 80% power and inflated for 10% lost-to-follow-up.

The estimated total number of participants for the adaptive design is 1223 ( *T. trichiura* 625, hookworm 312 and *S. stercoralis* 286). The sample size for the phase II component is 20% of the total participants for *T. trichiura* (126 participants), with the remaining 80% of *T. trichiura* participants randomized in the phase III component ( Table 1).

The study design has a blinded assessment for the primary endpoint, which is a laboratory-based measurement to be performed by blinded operators who will receive coded samples. Although a double-blind design would be methodologically superior, this would imply that the participants in two of the three treatment arms would have to swallow multiple placebo tablets, which could lead to unnecessary complications and interfere with the evaluation of several safety outcomes.

***Statistical analysis of the main outcomes***. For the safety analysis, an intent to treat (ITT) analysis (those participants who have received at least one dose of study intervention) will be used and participants will be considered by arm and by both FDC arms pooled (overall) and by number of doses of FDC received to explore dose-responses. First, all adverse events will be described and classified by causality, severity, seriousness and expectedness. Drug-related adverse events will be analyzed using ordinal logistic regression with the untoward effect classified as absent, mild, moderate, or severe and the factorial treatment regimens (without interaction term) as predictor variables. For a count outcome such as the number of adverse events or severe adverse events incidence rate ratio (IRR) and its 95% CI will be computed using Poisson or negative binomial regression.

The primary efficacy analysis will be based on the ITT population. Efficacy for each target STH species will be analyzed separately. A participant with multiple infections will be included in the analysis of each species that the participant is infected with. Cochran–Mantel–Haenszel (CMH) test, controlling the effect of site if that is appropriate (i.e., sufficient participants), will be used to compare the CRs for the three treatment groups. Differences among CRs will be assessed by using marginal modeling.

The difference in ERR between the different treatment arms will be assessed using three different approaches. First by an analysis of covariance (ANCOVA), in which the logarithm of the egg counts at post-treatment is the dependent variable, site (if appropriate) and treatment as fixed effect, and the logarithm of the egg count at pre-treatment will be used as the covariate. Baseline egg counts will also be analyzed as an independent variable that conditions treatment response. The second and third approaches will be to model ERR through marginal models and mixed models, respectively ^33^.

Correlation between Kato-Katz counts and qPCR C _t_ values will be explored through linear regression tests of Pearson's or Spearman´s (according to the underlying distribution). Kappa test will be used for the evaluation of both tests in the calculation of CRs. Relevant covariates will also be included in the data analysis.

All the analyzes will be carried out with the R software, packages geepack and lme4 ^26, 34, 35^.

***Anthelmintic resistance***. Anthelmintic resistance analysis will focus on *T. trichiura* and hookworms separately. In the case of hookworms (which comprise more than one species), anthelmintic resistance will be evaluated for each hookworm species if more than one species is present; and we predicted that *Necator americanus* will be the predominant species based on previous data ^23, 36^. Samples determined to be positive for these parasites by microscopy (pre- and post-treatment) will be included in the resistance evaluation and grouped by treatment arm.

To identify genetic variation associated with the treatment response, we will measure distribution of genome-wide genetic diversity between treatment groups and throughout the genome, consistent with recent studies on anthelmintic resistance human- and veterinary-infective helminth species ^37^. Genome-wide measures of within sample genetic diversity (for example, nucleotide diversity (pi), Waterson estimator, Tajima’s D, Fu’s) and between sample genetic diversity (e.g. *F*_ST_, Dxy, between pre- and post-treatment groups) will be estimated in non-overlapping sliding windows throughout the genome. Statistical significance will be inferred by the distribution of data points, from which data points that lie greater than three standard deviations from the genome-wide mean will indicate outliers of interest. We will identify genes in these outlier regions of genetic differentiation between parasite populations showing differences in treatment response (i.e., good responders vs poor responders, pre-treatment vs post-treatment) in the three treatment arms separately and in combination. We will also perform genome-wide association to identify variants in the parasite species associated with differences in treatment response and drug efficacy. Two different association tests will be performed: (i) a logistic regression genome-wide association between samples from participants with good treatment response phenotypes, and post-treatment samples from children with poor treatment response phenotypes; and (ii) a linear regression genome-wide association analysis with the ERR estimates as a continuous variable for all samples collected before treatment. We will characterize the predicted effects of genetic variation we uncover on genes and identify relationships between genes associated with the outlier variation by functional enrichment analyses.

***Pharmacokinetic analysis***. A simultaneous pharmacokinetic analysis of all concentration-time data of either IVM or ALB will be performed by means of the non-linear mixed effects modeling approach implemented in Phoenix program (Phoenix NLME version 8.2, Pharsight, RRID:SCR_003163. A Certara Company. Princeton, NJ, USA). The base model will be developed by fitting the one, two or three open compartment models with first-order elimination. Interindividual variability (IIV) will be tested in all the pharmacokinetic parameters assuming a log-normal distribution. Proportional and combined error will be applied to describe the residual error associated with the concentrations. Once the base model is developed, the effect of all covariates, physiologically reasonable (body weight, height, sex and age) and co-medication will be investigated on model parameters, graphically and by means of statistical multivariate models.

Covariates will be tested firstly univariate and then by the cumulative forward inclusion/backward elimination procedures. Significance levels of 5% (reduction in the minimum objective function value (MOFV) of >3.841 units) and 0.1% (increase in the MOFV of >10.8 units) will be employed during the forward addition and backward elimination steps. The decrease in the minimum objective function value (MOFV; -2xlog likelihood), parameter precision expressed as relative standard error (RSE%), reductions in IIV associated with a specific pharmacokinetic parameter, model completion status (e.g., successful convergence or termination) and visual inspection of goodness-of-fit plots will also be considered for model selection.

The final model evaluation will be performed through a prediction corrected visual predictive check to investigate the predictive capability of the model. Parameter precision will be assessed through a non-parametric bootstrap. Once the final model has been evaluated, simulations of different scenarios will be performed to establish the best dose for each body weight range of children.

Graphical diagnostics will be assessed using Phoenix version 8.2 or Xpose version 4.2.1 implemented in R version 3.6.0) ^26, 38^.

***Exploratory analyses***. Exploratory analysis will be performed to understand the potentially large number of unknown confounders/effect modifiers in this study. A bivariate analysis of baseline characteristics between included and excluded children will be performed. Subgroup safety and efficacy analyzes will also be performed in the ITT population. The subgroups to be analyzed will be:

- IVM drug exposure: categorized by >400 µg/Kg vs ≤400 µg/Kg.
- Age: categorized by SAC (5 to 14 years-old) and young adults (15 to 18 years-old).
- Co-infection: categorized by mono-infected vs co-infected.
- Worm burden: categorized by WHO categories of egg burden categories measured by Eggs Per Gram (EPG) through Kato-Katz method ^39^.

### Monitoring, ethics and regulatory

This study will be conducted in conformity with the principles set forth in the Declaration of Helsinki in its current version, the requirements of Good Clinical Practice (GCP) as defined in Guidelines and The International Council for Harmonization of Technical Requirements for Registration of Pharmaceuticals for Human Use (ICH) E6(R2), and all national and local regulations and guidance applicable at each site. Each institution engaged in this research will hold registered IRBs/ECs that will review and approve this protocol, associated informed consent documents, recruitment material, and handouts or surveys intended for the participants, prior to the recruitment, screening, and enrolment of participants. The IRB/IEC review shall be in accordance with ICH E6 (R2), and other regulations and policies, as applicable. The trial is registered at ClinicalTrials.gov, Identifier: NCT0512469; first posted on November 18, 2021.

The regulatory goal for the use of the FDC formulations (9 mg/400 mg and 18 mg/ 400 mg of IVM and ALB, respectively) against STH are subject to review and evaluation by the European Medicines Agency (EMA) for use outside the European Union through the EU-Medicines for all procedure “EU-M4all” (previously known as the Article 58 procedure). This program, operated by EMA in cooperation with WHO, provides scientific opinions on high priority human medicines, that are intended for markets outside of the European Union with the aims to facilitate prequalification of the medicine by WHO and registration in the target countries ^40^. As part of this process, the whole development of the products and the trial protocols have been discussed and adapted based on the results of three Scientific Advice rounds with EMA's Committee for Medicinal Products for Human Use (CHMP).

### Study status and timelines

The phase II component of the trial began recruitment of participants in February 2022 and is expected to progress to recruitment for the phase III component of the trial in all three field sites between May and July 2022 and complete recruitment by May 2023. Upon completion of the study, its results and interpretation will be shared through peer-reviewed, open-access scientific journals and at scientific conferences. A final study report will be presented to regulatory agencies and the data made available unless required to be concealed by IRBs or regulatory agencies.

## Discussion

This adaptive randomized clinical trial introduces an innovative combination of ALB and IVM with registrational goals and the aim of contributing to STH recommendations and control activities, as well as WHO prequalification. Besides STH, the safety, acceptability and pharmacokinetic data generated by this trial can also open possibilities of expanding its use to other NTD control programs currently using either or both drugs.

The main challenge in the development of a fixed combination of ALB and IVM was the different posology of both drugs; then, while ALB is well established with a single authorized fixed dosage of 400 mg for children older than 12 months as per WHO´s STH control guidelines ^7^, at present IVM has a flexible dosage regime of between 200 - 400 µg per kg body-weight. The objectives of the ALIVE trial and its design are supported by a development program carried by members of the STOP Project Consortium, that have demonstrated that IVM up to 600 µg/kg can be safely administered. This high dosage profile allows a fixed-dose, rather than commonly used weight or height based dosing, to be used here ^18^. Despite not strictly a high-dose regimen of IVM, since participants will receive a dose between 200- and 600 µg/kg of IVM, the data generated here will identify pharmacokinetic / pharmacodynamic parameters that will contribute towards an evidence-based therapeutic index for IVM in a combination regimen with ALB.

Existing limitations in diagnostic methodologies for the detection of STH in general and in particular for *S. stercoralis*, pose a significant challenge towards determining the efficacy of treatment in the context of a randomized clinical trial. In this context, a more precise assessment is required to identify meaningful significant differences between treatment arms, and are affected by sensitivity, specificity, reproducibility and operator dependency, the later emphasized in multicentric trials ^41^. The contribution of qPCR into solving these weaknesses will be explored in our trial and will add to efforts initiated by other groups to evaluate molecular biology tools as a potential improvement in the assessment of key outcomes in randomized clinical trials of anthelmintic drugs ^42^. While the current trial includes the implementation of this technology in all three clinical sites, the efficacy outcome will be processed in a centralized manner at KEMRI in Kilifi.

Although the drivers and mechanisms underlying anthelmintic drug resistance in STH of human interest are incompletely understood, there is extensive data from the veterinary world where it is a widespread problem ^43^. Moreover, a meta-analysis of randomized clinical trials against *T. trichiura* showed a temporal trend towards decreased efficacy of benzimidazole drugs and a recently published trial found lower efficacy of ALB in communities that have received MDA activities for more prolonged periods, suggesting drug resistance as a potential mechanism ^10, 44^. By using different mechanisms of action, ALB/IVM combinations provide regimens with a theoretical lower risk for the emergence of resistance. As in HIV and tuberculosis, co-formulations further contribute to it by ensuring compliance with a combination regimen. We also aim to identify markers of resistance through the comprehensive evaluation of pre- and post-treatment samples of *T. trichiura* through whole-genome sequencing. While the trial is powered for each STH species of interest ( *T. trichiura*, hookworms and *S. stercoralis*), it is powered for the entire study population rather than for each of the three study sites, which might constitute a limitation should significant differences in either or both treatment response and genetic differences unrelated to treatment response (i.e., population genetic differences) between study sites be found.

The trial also seeks to understand the potential benefit of three-day treatment regimens, which depart from the current dogma of single-dose regimens for MDA strategies against STH. While multiple-day regimens are in place for other indications affecting communities endemic for STH ^45^, the suboptimal efficacy of current regimens against *T. trichiura* calls for an evaluation of new approaches to improve the efficacy of treatment. To assess its feasibility in control programs, the trial includes palatability evaluations in the phase II trial. Acceptability evaluations will also be conducted in the communities and with program managers as an adjunct to the trials, which should shed light on the possibilities and limitations of three-day regimens and provide a comprehensive outlook into the potential impact of these regimens in the control and transmission interruption aims against STH.

From a programmatic perspective, a fixed-dose co-formulation provides advantages in packaging, transportation, storage and drug administration, each of which also positively impacts the cost of medical products and programs that deliver them.

## Conclusion

This trial seeks to provide evidence for the registration of a fixed-dose coformulation of ALB and IVM through the demonstration of its safety, efficacy and acceptability for the treatment of all STH species of interest for WHO-guided control activities. With an adaptive design, it will first define the safety of a FDC in phase II, followed by a phase III safety and efficacy trial. The data generated hopes to provide important advancements towards the safe and effective control of all STH in line with 2030 elimination of morbidity goals as a public health tool towards relieving the burden of STH on more than a billion people worldwide.

## Data availability

No data are associated with this article.
