# Supplementary material for: An adaptive phase II/III safety and efficacy randomized controlled trial of single day or three-day fixed-dose albendazole-ivermectin co-formulation versus albendazole for the treatment of Trichuris trichiura and other STH infections. ALIVE trial protocol
Source: Gates Open Res. 2022 May 5;6:62. [Version 1] doi: 10.12688/gatesopenres.13615.1 (PMC9714317; doi:10.12688/gatesopenres.13615.1)
Supplement: Peer review history

# Peer review: Reviewer response for version 1

**DOI:** 10.21956/gatesopenres.14889.r32202

**Published:** 2022-07-05

**Authors:** Kevin Kobylinski

**Affiliations:** 1 Department of Entomology, Armed Forces Research Institute of Medical Sciences, Bangkok, Thailand

This is an important trial which is well designed and will provide critical information for the use of a novel co-formulation of ivermectin and albendazole, which are likely the ideal drugs for soil-transmitted helminth control and elimination. There are a few points that could be explained better to make the trial design clearer and the rationale better justified. Dose escalation (and weights) for Phase II Groups 2 and 3 should be explained better in the Trial Design text, but figure 1 is helpful.

 It is not clear how many children in which weight class will be provided Arms 1, 2, or 3 as Table 2 suggests a 1:2:2 ratio distribution for treatment arms but there are different numbers listed for the Phase II weight classes in Figure 1, and the target weight distribution is not clearly explained for Phase III. Is there a rationale for performing Phase II Group 2 before Group 3? One assumes that the rationale is to evaluate the ivermectin dose range of 400-600 ug/kg in larger/older children prior to administering the same dose range 391-600 ug/kg to smaller/younger children, but it would be ideal to see rationale explained. The Phase II ivermectin pharmacokinetic output will provide excellent evidence for the effect of age on ivermectin bioavailablity. It has been shown that children <12 years old reach half the peak and total bioavailability of ivermectin compared to adults (Schulz *et al.,* 2019 ^1^) and two ivermectin pharmacokinetic models predict that higher doses of ivermectin are necessary in young children to reach the same levels as adults (Brussee *et al.,* 2019 ^2^, Gwee *et al.* 2020 ^3^). Some discussion as to the potential contribution of the ALIVE trial to understanding the influence of age on ivermectin pharmacokinetics in children is warranted.

Is the study design appropriate for the research question?

Yes

Is the rationale for, and objectives of, the study clearly described?

Yes

Are sufficient details of the methods provided to allow replication by others?

Yes

Are the datasets clearly presented in a useable and accessible format?

Not applicable

Reviewer Expertise:

Neglected Tropical Diseases, Entomology

I confirm that I have read this submission and believe that I have an appropriate level of expertise to confirm that it is of an acceptable scientific standard.

## References

[1] : Pharmacokinetics of ascending doses of ivermectin in Trichuris trichiura-infected children aged 2–12 years. *Journal of Antimicrobial Chemotherapy* .2019;74(6) : 10.1093/jac/dkz083 1642-1647 10.1093/jac/dkz083 PMC652448130859185

[2] : Ivermectin Dosing Strategy to Achieve Equivalent Exposure Coverage in Children and Adults. *Clin Pharmacol Ther* .106(3) : 10.1002/cpt.1456 661-667 10.1002/cpt.1456 30993667

[3] : Population pharmacokinetics of ivermectin for the treatment of scabies in Indigenous Australian children. *PLoS Negl Trop Dis* .14(12) : 10.1371/journal.pntd.0008886 e0008886 10.1371/journal.pntd.0008886 33284799PMC7746298

# Response

**Published:** 2022-08-11

**Authors:** ALEJANDRO JAVIER KROLEWIECKI

**Affiliations:** Universidad Nacional de Salta, Argentina

We thank Kevin Kobilinsky for his review and comments on our protocol, and take the opportunity to further clarify the following concepts:

 Dose escalation: among the study candidates of the Phase II trial, which recruits children between 15 and 45 Kg of body weight, the design is to start with the subgroup between 23 and 30 Kg, receiving doses of IVM between 300 and 391 µg/Kg and only upon DSMB clearance, to recruit the cohort including participants receiving a higher dose of IVM of 400 to 600 µg/Kg (children weighting 30 to 45Kg), followed by, after DSMB approval, the lighter group of children (15 to 23Kg), who received 391 to 600µ/Kg. The rationale for this study design is to progress to higher doses and younger/lighter participants progressively upon confirmation of adequate safety in each subgroup.

 Sample size in Phase II: in this trial, the 1:2:2 randomization between the 3 study arms is applied in the 3 sequential weight sub-groups; therefore within each sub-group the number of participants per study arms would be distributed approximately in a 1:2:2 manner.

 Weight distribution in Phase III: for this trial participants receive FDC 400/9mg if the weight is below 45Kg and 400/18 in those 45Kg and up, without any dose escalation included.

 PK: there is a scarcity of data on the pharmacokinetics of IVM in pediatric populations further worsened by an almost complete absence of evidence of PK/PD parameters related to antihelmintic drugs against STH. The effect of age on these parameters is another variable of consideration given variations in the expression of cytochrome P450s at different pediatric ages (1)

**References**

 1.         Upreti VV, Wahlstrom JL. Meta-analysis of hepatic cytochrome P450 ontogeny to underwrite the prediction of pediatric pharmacokinetics using physiologically based pharmacokinetic modeling. J Clin Pharmacol. 2016 Mar;56(3):266–83.

# Peer review: Reviewer response for version 1

**DOI:** 10.21956/gatesopenres.14889.r32064

**Published:** 2022-05-16

**Authors:** Antonio Montresor

**Affiliations:** 1 Department of Control of Neglected Tropical Diseases, World Health Organization, Geneva, Switzerland

The protocol is well written, complete and appropriate to reply to the research question. I have however some concerns on the relevancy of the research question:

 There are several studies that investigated the efficacy and safety of albendazole ivermectin co-administration (Hürlimann *et al.*, 2022 ^1^, Welsche *et al.*, 2021 ^2^, Keller *et al.*, 2021 ^3^, Palmeirim *et al.*, 2018 ^4^) and as a consequence, there is no doubt, in my opinion, that a combination of albendazole plus ivermectin (in Fixed Dose Combination or dosed according to weight) is more efficacious than albendazole alone against *T. trichiura* and *S. stercoralis *(on the other side, no additional efficacy of the Fixed Dose Combination should be expected on hookworms).

 In my opinion a much more relevant question is if the Fixed Dose Combination (that has clear advantages in terms of logistics and ease of administration) is as efficacious as albendazole and ivermectin dosed according to weight).

 In my opinion the protocol is a missed opportunity because it will provide already known replies and will not address an important research question that would allow simpliying the logistics of mass drug administration for the control of Soil Transmitted helminthiasis.

Is the study design appropriate for the research question?

Yes

Is the rationale for, and objectives of, the study clearly described?

Yes

Are sufficient details of the methods provided to allow replication by others?

Yes

Are the datasets clearly presented in a useable and accessible format?

Yes

Reviewer Expertise:

Neglected Tropical Diseases control

I confirm that I have read this submission and believe that I have an appropriate level of expertise to confirm that it is of an acceptable scientific standard.

## References

[1] : Efficacy and safety of co-administered ivermectin and albendazole in school-aged children and adults infected with Trichuris trichiura in Côte d'Ivoire, Laos, and Pemba Island, Tanzania: a double-blind, parallel-group, phase 3, randomised controlled trial. *Lancet Infect Dis* .22(1) : 10.1016/S1473-3099(21)00421-7 123-135 10.1016/S1473-3099(21)00421-7 34856181

[2] : Efficacy and safety of moxidectin and albendazole compared to ivermectin and albendazole co-administration in adolescents infected with Trichuris trichiura: a randomized controlled trial protocol. *Gates Open Res* .2021;5: 10.12688/gatesopenres.13299.2 106 10.12688/gatesopenres.13299.2 34632308PMC8488464

[3] : Long-term outcomes of ivermectin-albendazole versus albendazole alone against soil-transmitted helminths: Results from randomized controlled trials in Lao PDR and Pemba Island, Tanzania. *PLoS Negl Trop Dis* .15(6) : 10.1371/journal.pntd.0009561 e0009561 10.1371/journal.pntd.0009561 34191812PMC8277064

[4] : Efficacy and safety of co-administered ivermectin plus albendazole for treating soil-transmitted helminths: A systematic review, meta-analysis and individual patient data analysis. *PLoS Negl Trop Dis* .12(4) : 10.1371/journal.pntd.0006458 e0006458 10.1371/journal.pntd.0006458 29702653PMC5942849

# Response

**Published:** 2022-05-26

**Authors:** ALEJANDRO JAVIER KROLEWIECKI

**Affiliations:** Universidad Nacional de Salta, Argentina

Response to Dr. Montresor.

 We appreciate the comments and insightful opinions from Dr. Antonio Montresor on our protocol and would like to highlight that we fully agree that a regimen with improved efficacy against *T. trichiura* than what is achieved with albendazole is feasible through its combination with available medications, as is the case for ivermectin. Besides the available data on its efficacy, safety is also encouraging even at high doses of ivermectin, as demonstrated in preliminary trials performed by our groups in healthy adult volunteers and infected children (1,2).

 With the same ultimate implementation goals in mind as those expressed in the review, our Phase II/III adaptive trial is relevant and necessary. The ALIVE trial contains innovations in the strategy (a fixed-dose approach to the use of ivermectin along with albendazole) and in the product (the fixed-dose combinations (FDC)); as such, this is a registrational trial with primary safety and efficacy objectives. For that, the protocol has been developed with the scientific advice from the European Medicines Agency (EMA).

 The selection of albendazole as the control arm is based on the rationale of using the current standard of care as the proper comparator. The ALIVE trial is a key step in the development and registration of the FDC, which includes prior developments and trials as the acquisition of capacities for the industrial fermentation of ivermectin and the WHO prequalification of a generic ivermectin by Liconsa Labs, producer of the FDC and member of the STOP Consortium ( https://extranet.who.int/pqweb/news/lab-liconsa%E2%80%99s-ivermectin-prequalified), as well as trials showing the pharmacokinetic behavior of high dose ivermectin and the bioavailability of each component of the FDC in comparison to the reference products (manuscript under review); the later being the standard approach to assess the comparability of a drug combination to each of its components administered separately (1). As described in the manuscript, that bioavailability trial provided data supporting the design of the ALIVE trial.

 In full agreement with the opinion of Dr Montresor regarding the logistic advantages foreseen for the FDC, the STOP project includes further studies assessing the acceptability and implementation strategy for these innovations.

 References

1. Muñoz J, Ballester MR, Antonijoan RM, Gich I, Rodríguez M, Colli E, et al. Safety and pharmacokinetic profile of fixed-dose ivermectin with an innovative 18mg tablet in healthy adult volunteers. Prichard RK, editor. PLoS Negl Trop Dis. PLoS Negl Trop Dis; 2018;12:e0006020.
2. Matamoros G, Sánchez A, Gabrie JA, Juárez M, Ceballos L, Escalada A, et al. Efficacy and Safety of Albendazole and High-Dose Ivermectin Coadministration in School-Aged Children Infected With Trichuris trichiura in Honduras: A Randomized Controlled Trial. Clin Infect Dis; 2021;73:1203–10.Response to Dr. Montresor.
